# Acceptability of cannabidiol in patients with psychosis

**DOI:** 10.1177/20451253221128445

**Published:** 2022-10-25

**Authors:** Edward Chesney, Doga Lamper, Millie Lloyd, Dominic Oliver, Emily Hird, Philip McGuire

**Affiliations:** Department of Psychosis Studies, Institute of Psychiatry, Psychology and Neuroscience, King’s College London, London, SE5 8AF, UK; Department of Psychosis Studies, Institute of Psychiatry, Psychology and Neuroscience, King’s College London, London, UK; Department of Psychosis Studies, Institute of Psychiatry, Psychology and Neuroscience, King’s College London, London, UK; Department of Psychosis Studies, Institute of Psychiatry, Psychology and Neuroscience, King’s College London, London, UK; Department of Psychosis Studies, Institute of Psychiatry, Psychology and Neuroscience, King’s College London, London, UK; Department of Psychosis Studies, Institute of Psychiatry, Psychology and Neuroscience, King’s College London, London, UK

**Keywords:** acceptability, cannabidiol, CBD, Psychosis, Schizophrenia

## Abstract

**Background:**

Cannabidiol (CBD) is a promising novel candidate treatment for psychosis. It has a more benign side effect profile than antipsychotic medications, and being treated with CBD is not perceived as being stigmatising. These observations suggest that patients with psychosis would find CBD to be a relatively acceptable treatment.

**Objective:**

This study tested the above hypothesis by assessing the views of a sample of patients.

**Methods:**

Patients with a psychotic disorder were invited to complete a survey exploring their expectations about the efficacy and side effects of CBD.

**Results:**

Seventy patients completed the survey. The majority (86%) were willing to try CBD as a treatment. Most patients believed that CBD would improve their psychotic symptoms (69%) and that it would have fewer side effects than their current medication (64%; mainly antipsychotics). A minority of patients (10%) were concerned that CBD might exacerbate their psychotic symptoms. This, however, appeared to reflect confusion between the effects of CBD and those of cannabis.

**Conclusion:**

Most patients with psychosis regard CBD as an acceptable treatment. Although CBD has not yet been approved as a treatment for psychosis, many patients are aware of it through the presence of CBD in cannabis and in health supplements. When added to the emerging evidence of its efficacy and the low risk of side effects, the high acceptability of CBD underlines its therapeutic potential.

## Introduction

Antipsychotic medications are the main treatment for psychotic disorders. Although they can reduce psychotic symptoms,^1^ many patients are reluctant to take them because of side effects like weight gain, metabolic syndrome, sedation, sexual dysfunction, and extrapyramidal symptoms.^2–5^ In addition, because antipsychotic drugs are associated with severe mental illnesses, particularly schizophrenia, patients may be deterred from taking them because of concerns about stigmatisation.^6^ The acceptability of antipsychotic medications in patients with psychosis is thus relatively poor and contributes to the low levels of adherence to recommended treatment.^7^ This has major clinical consequences, as it increases the risk of relapse and functional deterioration, and is associated with reduced life expectancy.^8–10^

Cannabidiol (CBD) is a naturally occurring phytocannabinoid produced by the cannabis plant. Data from case series and small-scale clinical trials suggest that it has efficacy in the treatment of psychosis.^11–16^ CBD may also protect against the harmful effects of cannabis use.^17,18^ The mechanism of action through which it produces these putative effects has not been established, but may include the inhibition of endocannabinoid metabolism and/or activity at cannabinoid and serotonergic receptors.^11,19–21^

Data from studies in patients with psychosis and in other patient groups indicate that CBD has few side effects. A meta-analysis of randomised, placebo-controlled trials found that the only side effect directly attributable to CBD was diarrhoea.^22^ Other adverse effects, however, may arise from CBD inhibiting cytochrome P450 enzymes, leading to drug–drug interactions, for example, with benzodiazepines.^22^ This relatively benign side effect profile contrasts with that of antipsychotic medications.^23^ Moreover, CBD is not linked with the stigma that can be associated with taking antipsychotics. This may be because it is derived from cannabis and because it is already available in over-the-counter health supplements.^24,25^

These observations suggest that CBD may have a relatively high level of acceptability as a treatment among patients with psychosis. Its acceptability as a treatment in this group, however, has yet to be formally assessed. The aim of this study was to address this issue by conducting a survey in patients with psychosis. Our main hypothesis was that CBD would have a high level of acceptability as a treatment.

## Methods

Data were obtained from a clinical audit of over-the-counter CBD use in patients with psychosis. The audit was approved according to local procedures at the South London and Maudsley NHS Foundation Trust, London, UK (REF: Service Evaluation Audit P300). It therefore did not require research ethics committee approval. Non-random convenience sampling was used to recruit patients receiving secondary mental health care from community and inpatient services for patients with psychosis. Participants were approached by the interviewers between August 2021 and February 2022.

The inclusion criteria were the following:

- Diagnosis of a psychotic disorder or bipolar disorder, as defined by the *International Classification of Diseases* version 10 (F20-F29, F31)
- Receiving secondary mental healthcare
- Providing verbal consent and demonstrating adequate mental capacity
- Stable mental state

Researchers provided patients with a short verbal description of the study and explained that participation was voluntary and anonymous. All participants provided informed verbal consent. A structured questionnaire was used to collect data on demographic and clinical variables, cannabis use, the participant’s knowledge of CBD and any previous experience with CBD (in any form).

If a participant did not know what ‘Cannabidiol’ or ‘CBD’ was, the researcher provided them with a standardised verbal description. This stated that cannabidiol/CBD was derived from cannabis, is available as a health supplement and is being tested as a new treatment for psychosis.

Participants were then asked the following questions:

### Treatment effects

1. If CBD was available as a medication to treat your illness, would you want to take it? [Yes, No] [if no, specify]
2. Compared with now, I think my psychosis symptoms after taking CBD would be. . . [a lot worse, slightly worse, somewhat worse, no different, slightly better, somewhat better, a lot better]

### Side effects

3. Do you think CBD has any side effects? [Yes, No] [if yes, specify]
4. Compared with the side effects I get from my current medication, I think the side effects with CBD would be. . . [a lot worse, slightly worse, somewhat worse, no different, slightly better, somewhat better, a lot better]

### Formulation

5. Would you be happy to take CBD as a. . . [tablet, capsule, oil, other]
6. Which form of CBD would you prefer to take?

### Statistical analysis

Willingness to trial CBD was analysed according to demographic and clinical variables, tested with Fisher’s exact test. Fisher’s exact test was used as the expected values in contingency tables were small. When there were more than two categories, a pairwise Fisher’s exact test was used to examine all possible contrasts. The threshold for statistical significance was set as *p* < 0.05. All analyses were completed using R version 3.6.3.

## Results

The demographic and clinical characteristics of sample (*n* = 70) are shown in Table 1.

**Table 1.** The proportion of participants who would consider taking CBD as a treatment for their illness according to demographic and clinical characteristics.

		***n* (%)**	**Would consider using CBD as a treatment**	
			Yes (%)	*p* value
	Total sample	70 (100)	60 (86)	
**Sex**	Male	46 (66)	43 (93)	
	Female	24 (44)	17 (71)	0.03
Age	<35 years	33 (47)	30 (91)	
	⩾35 years	37 (53)	30 (81)	0.32
Ethnicity	White	19 (27)	16 (84)	
	Black Caribbean	21 (30)	19 (90)	
	Black African	19 (27)	16 (84)	
	Other	11 (16)	9 (82)	0.85
Diagnosis	Schizophrenia (F20)	32 (46)	26 (81)	
	Affective psychoses (F25 & F31)	15 (21)	13 (87)	
	Other psychoses (F23 & F29)	23 (33)	21 (91)	0.68
Illness stage	First episode psychosis	35 (50)	31 (89)	
	Established illness	35 (50)	29 (83)	0.73
Cannabis use	Current user	20 (29)	20 (100)	
	Previous user	27 (39)	27 (100)	
	Never user	23 (33)	13 (57)	<0.00001*
Aware of CBD	Yes	46 (66)	40 (87)	
	No	24 (34)	20 (83)	0.73
Ever used a CBD product	Yes	20 (29)	20 (100)	
	No	50 (71)	40 (80)	0.053

CBD: cannabidiol.

* Both current and previous cannabis users had significantly higher rates of acceptability compared to never users (*p* < 0.001).

All of the patients had a psychotic disorder: 79% had a diagnosis of schizophrenia or another non-affective psychosis, and 21% had an affective psychosis diagnosis. The mean age was 38 years and 66% of the patients were male. Most (66%) had already heard of CBD, 29% had used an over-the-counter CBD product, but none had taken pharmaceutical-grade CBD. Most participants (67%) had previously used cannabis. Almost all (94%) of the sample were being treated with an antipsychotic medication (72% oral; 28% long-acting injectable). About 22% of the sample were taking a mood stabiliser and 11% were taking an antidepressant.

The majority of participants (86%) were willing to consider using CBD as a treatment for their disorder (Table 1). All participants who were current or past users of cannabis were willing to try treatment with CBD, compared with only 57% of never cannabis users (*p* < 0.001 for each comparison). Similarly, all participants who had previously used over-the-counter CBD products were willing to try treatment with CBD, compared with 80% of those who had never used them. Male participants were more likely to consider using CBD than females, but there were no differences between younger and older patients, or between patients from different ethnic groups. Table 1 summarises the demographic and clinical characteristics which were associated with this response.

Only 10 participants indicated that they would not use CBD as a treatment. Their reasons included that it might harm their mental health (*n* = 3), lack of evidence for its efficacy (*n* = 2), it is derived from cannabis (*n* = 2), and fear of intoxication (*n* = 1). One participant declined to give a reason and one said that they did not need any type of treatment.

**Figure 1.**
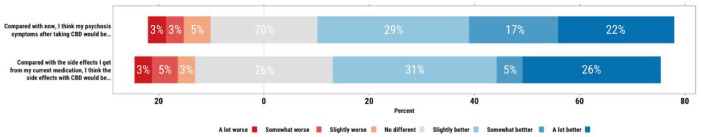
Participants’ expectations regarding the efficacy and side effects of CBD.

Fifty-eight patients indicated how they thought CBD treatment would affect their psychotic symptoms (Figure 1). Forty (69%) thought that their symptoms would improve, while seven (12%) thought it would make them worse.

### Side effects

Fifty-nine participants answered questions about side effects. Twenty-eight (47%) said that they thought that CBD would not have any side effects. When asked to compare CBD with their current medication, 38 participants (64%) thought that CBD would have fewer side effects, while seven (12%) thought that it would have more side effects (Figure 1). The side effects anticipated for CBD included sedation (*n* = 11), exacerbation of psychotic symptoms (*n* = 8), gastrointestinal symptoms (*n* = 2), headache (*n* = 2), hunger (*n* = 1), addiction (*n* = 1), incontinence (*n* = 1), and impaired liver function (*n* = 1).

### Formulation

**Figure 2.**
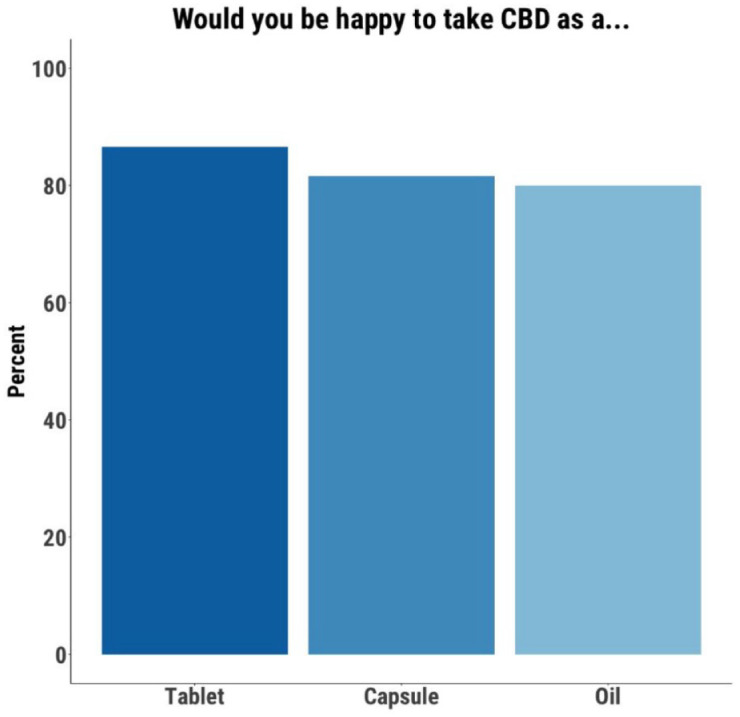
Tablet, capsule and oil formulations are acceptable to most participants.

**Figure 3.**
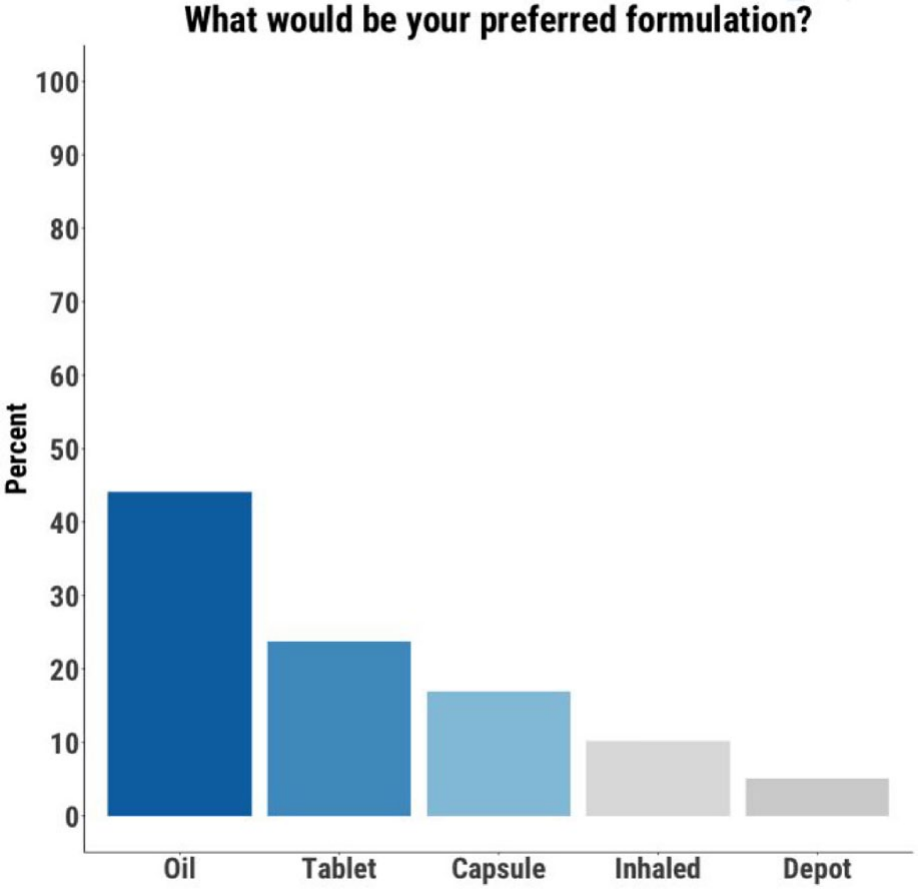
Oil was the preferred formulation for the largest proportion of participants.

Those open to using CBD were asked about the acceptability of different formulations. Of the 59 participants who provided a response, 52 (88%) indicated that they would be open to using a tablet, 49 (83%) a capsule, and 48 (81%) an oil (Figure 2). When asked for their preferred formulation, 26 participants (44%) chose an oil, 14 (24%) chose a tablet, 10 (17%) chose a capsule, six (10%) chose a vape/cigarette, and three (5%) chose an injectable/depot formulation (Figure 3).

## Discussion

Although anecdotal reports suggest that CBD is viewed relatively positively by patients with psychotic disorders as a potential treatment, there is surprisingly little evidence for this. This study sought to address this issue by conducting a survey among patients with psychotic disorders in secondary care. Our findings indicate that for most patients with psychosis CBD is an acceptable treatment.

At present, although there is preliminary evidence that CBD may be effective in psychosis,^14,15^ it is not yet approved as a treatment. Although none of the patients who completed our survey had ever taken pharmaceutical-grade CBD, most had heard of CBD and 29% had used an over-the-counter product containing it. In addition, 67% of the sample had previously used cannabis which may have contained CBD. CBD is thus an unusual novel candidate treatment, in that the patient population already has a high degree of familiarity with it. This is likely to increase the willingness of patients to participate in clinical trials of CBD and, if it is subsequently licenced as a medication, to accept it as a treatment for psychosis. In contrast, many patients with psychosis are reluctant to take antipsychotic medications, which have a low level of acceptability as a treatment. As a result, adherence to treatment with these drugs is often poor^7^ and this increases the risk of adverse clinical outcomes, such as acute relapse, hospital admission, functional decline and mortality.^8–10^

As well as being willing to accept CBD as a treatment, most respondents thought that it would improve their symptoms and would be associated with fewer side effects than their current medication. Indeed, almost half of the sample thought that CBD would have no side effects at all. These results show that patients’ positive expectations about CBD extend to both its therapeutic and its adverse effects. In contrast, many patients have a negative view of antipsychotic medications. In one survey of 212 patients with schizophrenia, 46% said they had a ‘negative’ or ‘very negative’ opinion of antipsychotics compared with 42% who said they had a ‘positive’ or ‘very positive’ opinion of them.^26^ In another survey, 31 of 61 patients (51%) said they had a positive view of antipsychotic medications; the majority of this group (20/31), however, expressed ‘distinct reservations’.^27^ Expectations about treatment have been shown to modify the clinical response to treatments for depression, anxiety and pain^28–30^ and may contribute to the placebo effect, which can be significant in patients with psychosis.^31^ It is thus possible that the relatively positive expectations about treatment with CBD will enhance its effectiveness in real-world practice, by augmenting its therapeutic effects and diminishing adverse events.

Non-psychotic symptoms, such as irritability, insomnia, anxiety and low mood, are often the first signs of a psychotic relapse^32^ and are also features of the clinical high risk state that precedes the onset of psychosis.^33^ As these symptoms are also among the most common lay indications for the use of over-the-counter CBD products,^25^ patients with psychosis may also be inclined to accept CBD as a treatment because they think it will help with these other symptoms.

Most of the participants in our sample were past or current cannabis users. This is in keeping with the generally high prevalence of cannabis use among patients with psychosis.^34,35^ In this study, a history of cannabis use was the strongest predictor of willingness to consider treatment with CBD: 100% of cannabis users were open to CBD, compared with 57% of never users. Among patients with psychosis, those with co-morbid cannabis use have more severe symptoms and an increased risk of relapse,^36^ which is partly attributable to relatively poor adherence to antipsychotic medications.^37^ The very high acceptability of CBD in these patients suggests that it could be particularly useful as a treatment in this subgroup. Moreover, independent research indicates that CBD can counteract the pro-psychotic effects of cannabis, which are mediated through delta-9-tetrahydrocannabinol.^17,18^ CBD might thus have a dual benefit in these patients, acting as an antipsychotic and reducing the adverse effects of concurrent cannabis use.^38^

The participants who were least willing to consider treatment with CBD were those who had never used cannabis. The most common concerns were that CBD would exacerbate their psychotic symptoms or cause sedation. These patients may have been confusing the effects of CBD with those of cannabis and this finding highlights the importance of educational work on the distinctions between the two. In particular, in contrast to cannabis, CBD is legal; is not intoxicating, sedative or addictive; and may reduce psychotic symptoms rather than exacerbate them. It is also important that patients are aware of the difference between pharmaceutical-grade and over-the-counter CBD products. Pharmaceutical-grade medicines are highly purified and usually contain CBD at considerably higher doses than is present in over-the-counter products. Moreover, some over-the-counter products have been found to contain significant levels of delta-9-tetrahydrocannabinol and other compounds that could have unwanted effects.^24^

One limitation of this study is the sampling strategy. As a non-random convenience sampling strategy was used, there is a risk that the sample may not be representative of the overall population of people with psychotic disorders. Of note, the majority of the participants had used cannabis previously. Although the prevalence of cannabis use in our patient sample is similar to that described in other samples from large urban areas with a diverse population, the prevalence is often lower in other regions.^35,39^ In this study, because a high proportion of the patients had used cannabis, and this was strongly associated with a willingness to try CBD, the acceptability may be higher than it would be in samples recruited from areas where cannabis use among patients is less common. Our sample only included patients who had agreed to participate in the survey and therefore may not be representative of all patients, as it may have been less likely to include those who had not engaged well with mental health services. We did not record the number of patients who did not agree to participate in the survey, so the potential extent of this issue is unclear. A further limitation was that when participants were asked about their willingness to be treated with CBD, the survey did not specify whether this was as a monotherapy or as an adjunct to antipsychotic medication.

Importantly, to our knowledge, this is the first ever study to assess the acceptability of CBD as a treatment in psychosis. When added to the emerging evidence that CBD has therapeutic effects and a low risk of side effects, this apparently high level of acceptability highlights the potential value of CBD as a novel treatment.

## Declarations

**Ethics approval and consent to participate:** The audit was approved by the South London and Maudsley NHS Foundation Trust, London, UK (REF: Service Evaluation Audit P300). All participants provided informed verbal consent that was recorded as part of the inclusion criteria assessment.

**Consent for publication:** Not applicable.

**Availability of data and materials:** Data and materials may be made available on request after approval from South London and Maudsley NHS Foundation Trust.
